# 肺癌患者围手术期预防性抗凝的临床价值分析

**DOI:** 10.3779/j.issn.1009-3419.2018.10.06

**Published:** 2018-10-20

**Authors:** 慧 徐, 虎 廖, 国卫 车, 坤 周, 梅 杨, 伦旭 刘

**Affiliations:** 610041 成都，四川大学华西医院胸外科 Department of Thoracic Surgery, West China Hospital, Sichuan University, Chengdu 610041, China

**Keywords:** 肺肿瘤, 围手术期预防性抗凝, 肺栓塞, Lung neoplasms, Perioperative prophylactic anticoagulation, Pulmonary embolism

## Abstract

**背景与目的:**

肺癌患者围手术期肺栓塞（pulmonary embolism, PE）发生率有增高的趋势，预防肺栓塞是加速康复外科的重要组成部分，然而肺癌患者围术期预防性抗凝时机仍存在争议。本研究旨在探讨肺癌患者围手术期应用抗凝药物预防肺栓塞的安全性和有效性。

**方法:**

连续收集2016年6月-2016年12月，在四川大学华西医院胸外科行胸腔镜肺解剖性肺切除术的肺癌患者共562例，其中56例肺癌患术前12 h开始应用低分子肝素（low molecular weight heparin, LMWH）抗凝直到出院；506例患者术后24 h开始应用直到出院。分析围手术期胸腔引流量、术后出血和肺栓塞发生率、肺部相关并发症等。

**结果:**

① 凝血酶原时间（prothrombin time, PT）、活化部分凝血活酶时间（activated partial thromboplastin time, APTT）和国际标准化比值（international normalized ratio, INR）在术前[(11.5±3.9) s, (27.8±3.5) s, (0.96±0.06) s]与术后抗凝组[(11.4±1.4) s, (28.3±4.0) s, (0.98±0.07) s]均无统计学差异（*P*=0.796, *P*=0.250, *P*=0.137）；Caprini评分在术前（3.1±1.8）和术后（3.3±1.5）抗凝组也无统计学差异（*P*=0.104）；②麻醉时间和术中出血量在术前抗凝组[(130.2±53.9) min, (76.8±49.3) mL]和术后抗凝组[(142.2±56.5) min, (73.7±41.6) mL]均无统计学差异（*P*=0.067, *P*=0.201)。③术后72 h总引流量在术前抗凝组[(728.1±505.7) mL]显著高于术后抗凝组[(596.4±373.5) mL]（*P*=0.005），而两组患者术后总引流量[(1, 066.8±1, 314.6) mL, (907.8±999.8) mL]无差异（*P*=0.203）；④肺栓塞和术后出血发生率在术前（1.785%, 1.785%）和术后（0.019%, 0.039%）抗凝组均无显著性差异（*P*=0.525, *P*=0.300）；⑤皮下气肿和肺部感染发生率在术前（1.785%, 14.285%）和术后（1.581%, 6.324%）抗凝组均无显著性差异（*P*=0.989, *P*=0.085）。

**结论:**

肺癌患者术前或术后预防性应用抗凝药物临床效果相当。

## Abstract (en)

**Background and objective:**

The incidence of pulmonary embolism (PE) in lung cancer patients who underwent surgery increased during the perioperative period, and prophylactic anticoagulation therapy was important part of enhanced recovery after surgery (ERAS). However, the timing of preventive anticoagulation in patients with lung cancer remained controversial. This study was designed to investigate the safety and efficacy of perioperative prophylactic anticoagulation therapy for lung cancer patients.

**Methods:**

Retrospective research was conducted on 562 lung cancer patients who underwent video-assisted thoracoscopic (VATS) anatomic pulmonary resections in West China Hospital from June 2016 to December 2016. 56 patients were treated with low molecular weight heparin (LMWH) anticoagulation 12 hours before operation until discharge, while the other 506 patients were treated with LMWH 24 hours after operation until discharge. The postoperative chest drainage volume, postoperative bleeding, pulmonary embolism incidence, and respiratory complications were analyzed.

**Results:**

(1) There were no significant differences in prothrombin time (PT), activated partial thromboplastin time (APTT) and international normalized ratio (INR) between the pre-operation prophylactic anticoagulation group (PRE group) [(11.5±3.9) s, (27.8±3.5) s, (0.96±0.06) s] and the post-operation prophylactic anticoagulation group (POST group) [(11.4±1.4) s, (28.3±4.0) s, (0.98±0.07) s] (*P*=0.796, *P*=0.250, *P*=0.137), and there was no significant difference in Caprini score between the PRE group (3.1±1.8) and the POST group (3.3±1.5) (*P*=0.104). (2) There were no significant differences in anesthesia time and intraoperative bleeding between PRE group [(130.2±53.9) min, (76.8±49.3) mL] and POST group [(142.2±56.5) min, (73.7±41.6) mL] (*P*=0.067, *P*=0.201). (3) The total drainage volume in 72 hours after operation in PRE group [(728.1±505.7) mL] was significantly higher than that of POST group [(596.4±373.5) mL] (*P*=0.005), while there were no significant differences between the two groups in total postoperative drainage volume [(1, 066.8±1, 314.6) mL *vs* (907.8±999.8) mL, *P*=0.203]. (4) There were no significant differences between the two groups in pulmonary embolism incidence (1.785% *vs* 0.019%, *P*=0.525) and postoperative bleeding rates (1.785% *vs* 0.039%, *P*=0.300). (5) There were no significant differences between PRE group and POST group in subcutaneous emphysema incidence (1.785% *vs* 1.581%, *P*=0.989) and pulmonary infection rates (14.285% *vs* 6.324%, *P*=0.085).

**Conclusion:**

The clinical value of preoperative prophylactic anticoagulation is equal to postoperative prophylactic anticoagulation for lung cancer patients.

外科治疗仍是肺癌患者根治的主要手段，而术后的加速康复需要有效预防术后并发症的发生，尤其是肺栓塞^[1, 2]^。肺栓塞发生的危险因素：一是肺癌手术时肺动脉内壁挫伤及动脉断端血流状态改变，容易形成血栓；二是术后由于疼痛及各种管道束缚，限制了患者早期下床活动^[3]^。肺癌术后肺栓塞发生率为0.18%-17.6%^[4-6]^，是导致肺癌患者围术期死亡的重要因素之一。研究^[7-9]^表明，预防性抗凝可以显著减少恶性肿瘤患者术后静脉血栓栓塞症（venous thromboembolism, VTE）的发生。目前肺癌手术患者预防性抗凝开始的时机仍存在争议。国内外不少指南推荐高危患者术前2 h-12 h开始抗凝^[10-12]^，也有研究发现术前预防性使用药物抗凝并没有降低术后VTE相关围术期死亡，但术后出血风险显著升高^[7]^。为研究肺癌患者围手术期预防性抗凝药物的有效性和安全性，我们分析了术前和术后应用抗凝药物的临床应用效果，结果如下。

## 1 对象与方法

### 1.1 研究对象

连续性分析四川大学华西医院胸外科2016年6月1日-2016年12月31日行胸腔镜解剖性肺手术的肺癌患者711例，纳入标准：①年龄19岁-75岁；②病理学检查诊断为原发性肺癌；③手术方式肺叶或肺段切除术+系统淋巴结清扫术；④临床资料完整且签署知情同意书；⑤无出血性疾病或术前服用抗凝药病史。排除标准：①病理诊断为转移性肺癌患者或病历资料不完整；②未签署知情同意书的患者；③术前服用抗凝类药物等；④术后出血或持续漏气需要再次手术的患者。最终纳入患者562例，男208例，女354例；腺癌390例，非腺癌172例。Ⅰ期390例，Ⅱ期70例，Ⅲ期+Ⅳ期102例，术后分期采用国际抗癌联盟（Union for International Cancer Control, UICC）（第八版）肺癌分期标准。其中术前抗凝组（pre-operation prophylactic anticoagulation group, PRE group）56例，术后抗凝组（post-operation prophylactic anticoagulation group, POST group）506例。所有患者术前均行VTE预防宣教及Caprini评分^[13]^，根据评分结果对患者VTE危险性进行分级，中度危险（3分-4分）及高度危险（≥5分）的患者分别用黄色及红色标识牌在床头标识（表 1）。

**1:** 两组患者的临床资料 The clinical demographic characters of study subjects

Characteristics	POST group (*n*=506)	PRE group (*n*=56)	*P*
Gender			0.832
Male	188 (37.1%)	20 (35.4%)	
Female	318 (62.9%)	36 (64.6%)	
Age (Mean±SD, yr)			
Men	55.3±14.3	57.8±10.5	0.103
Female	61.6±7.5	59.7±11.1	0.232
High (Mean±SD, cm)			
Men	165.7±10.8	165.5±12.6	0.160
Female	154.5±13.1	156.3±9.5	0.083
Body weight (Mean±SD, kg)			
Men	65.0±11.8	64.8±12.2	0.323
Female	55.6±10.8	57.8±11.6	0.143
Smoking status			
Never	335 (66.3%)	40 (71.7%)	0.127
Current/ever	171 (33.7%)	16 (28.3%)	0.216
Comorbidities			
Hypertension	187 (36.9%)	21 (37.5%)	0.309
Diabetes mellitus	119 (23.5%)	10 (18.8%)	0.062
Surgical approach			
Lobectomy	396(78.3%)	41 (72.5%)	0.324
Segmentectomy	110(21.7%)	15 (27.5%)	0.311
Histology			
Adenocarcinoma	354 (69.9%)	36 (63.8%)	0.928
Non-adenocarcinoma	152 (30.1%)	20 (34.1%)	0.233
TNM stage			
Stage Ⅰ	331 (65.5%)	39 (70.2%)	0.529
Stage Ⅱ	65 (12.9%)	5 (8.7%)	0.067
Stage Ⅲ+Ⅳ	110 (21.6%)	12 (21.1%)	0.424
Anesthesia time (Mean±SD, min)	130.2±53.9	142.2±56.5	0.067
Volume of blood loss (Mean±SD, mL)	76.8±49.3	73.7±41.6	0.201
Caprini score	3.1±1.8	3.3±1.5	0.104
Preoperation PT (Mean±SD, s)	11.5±3.9	11.4±1.4	0.796
Preoperation APTT (Mean±SD, s)	27.8±3.5	28.3±4.0	0.250
Preoperation INR	0.96±0.06	0.98±0.07	0.137
PT: prothrombin time; APTT: activated partial thromboplastin time; INR: international normalized ratio.			

### 1.2 方法

#### 1.2.1 手术方法

采用单向式胸腔镜肺叶或肺段切除法^[14]^。肺癌患者需行系统淋巴结清扫，左侧必须清扫第5、6、7、8、9、10组淋巴结，右侧包括第2、3、4、7、8、9、10组淋巴结^[15]^。引流管应用方法：28F胸腔引流管（扬州市邗江华飞医疗器件厂）所有患者术后均应用单引流管是将28 F引流管从第7肋间镜孔置入胸腔内，28 F常规从后胸壁放在胸顶，线结固定和留置线^[16-18]^，两组患者术后均采用相同的水封引流瓶，且均不加用负压吸引。

#### 1.2.2 预防性抗凝方法

肺癌患者术前评分中危以上（≥3分）的患者，术前抗凝组（PRE组）于术前12 h，术后抗凝组（POST组）于术后当天，均使用低分子肝素0.4 mg/d，两组患者均应用至出院当天。若术后出现引流液为血性液体，诊断为出血，则停用低分子肝素。所有患者均要求术后24 h内下床活动。

#### 1.2.3 镇痛方法

术后两组患者均采用静脉自控镇痛泵（patient controlled intravenous analgesia, PCIA）为基础镇痛方式，镇痛泵内药物为芬太尼、曲马多、托烷司琼和生理盐水配至100 mL，持续剂量2 mL/h。

#### 1.2.4 术后处理

拔管后均鼓励患者咳嗽，必要时刺激患者咳嗽^[19]^。术后第1天均行胸部X线片检查，若无肺漏气且每天引流量小于300 mL，肺已复张则拔除引流管。术后均早期促使患者下床活动。镇痛泵于引流管拔除的同时也一起停止。术后当天进食流质饮食，之后进食普食。

### 1.3 观察指标

#### 1.3.1 肺栓塞

根据中华医学会肺血管病组发布《中国急性肺栓塞诊断与治疗指南》和ESC 2014年急性肺栓塞诊断和管理指南，高度怀疑急性肺栓塞和经胸部双源CT或肺动脉造影确诊^[20]^。

#### 1.3.2 胸腔引流量

术后72 h引流量是指术后72 h的总引流量；术后总引流量是指术后总的引流量。

#### 1.3.3 术后出血

术后出血是指术后胸腔流突然增加，且呈血性，停用低分子肝素后，引流液减少，且不需手术止血治疗。

#### 1.3.4 乳糜胸

本研究诊断标准：手术后非血性引流液24 h引流量≥500 mL，且持续≥3 d；禁食治疗后治愈且不需要手术处理。

#### 1.3.5 皮下气肿

① 超过手术侧胸壁的皮下积气；②需要作皮肤切口引流；③皮下积气≥15 d。

#### 1.3.6 肺部感染^[21]^

含有以下指标3个或以上的应视为手术后肺炎：①手术后72 h的发热，体温 > 38 ℃；或72 h以内的体温再度升高。②白细胞计数升高（> 12×10^9^/L-15×10^9^/L）或白细胞计数回复正常值以后的再度升高，超过10×10^9^/L。③胸部影像学提示肺组织实变或不断增加的斑片状阴影。④咳出脓性痰液，或痰培养阳性。其中如果包含④，仅需要其他一项即可视为术后肺炎。呼吸科会诊确定为肺部感染，并需要更换抗生素或延长抗生素使用时间。

#### 1.3.7 麻醉时间

从气管开始到患者清醒拔除气管插管。

#### 1.3.8 术后住院日

手术当天到出院当天时间。

### 1.4 统计学分析

统计分析采用SPSS 16.0软件处理，计数资料采用实际例数及百分比表示，计量资料采用均数±标准差（Mean±SD）表示。计数资料的比较采用χ^2^或MonteCarlo确切概率法进行分析，正态分布计量资料比较采用两独立样本的*t*检验，非正态分布计量资料采用秩和检验法，双侧检验，*P* < 0.05为差异有统计学意义。

## 2 结果

### 2.1 两组肺癌患者术前及术中相关资料分析

PT、APTT和INR在术前抗凝组[(11.5±3.9) s, (27.8±3.5) s, (0.96±0.06) s]与术后抗凝组[(11.4±1.4) s, (28.3±4.0) s, (0.98±0.07) s]均无统计学差异（*P*=0.796, *P*=0.250, *P*=0.137）；Caprini评分在术前（3.1±1.8）和术后（3.3±1.5）抗凝组也无统计学差异（*P*=0.104）；麻醉时间和术中出血量在术前抗凝组[(130.2±53.9) min, (76.8±49.3) mL]和术后抗凝组[(142.2±56.5) min, (73.7±41.6) mL]均无统计学差异（*P*=0.067, *P*=0.201)。

### 2.2 两组肺癌患者术后相关临床指标分析

术后72 h总引流量在术前抗凝组[(728.1±505.7) mL]显著高于术后抗凝组[(596.4±373.5) mL]（*P*=0.005），而两组患者术后总引流量[(1, 066.8±1, 314.6) mL, (907.8±999.8) mL]无差异（*P*=0.203）；引流管留置时间和术后住院日在术前[(4.4±4.6) d, (6.8±6.7) d]和术后[(4.1±3.0) d, (6.2±3.7) d]抗凝组均无显著性差异（*P*=0.405, *P*=0.196）。

### 2.3 两组肺癌患者围手术期并发症分析

肺栓塞和术后出血发生率在术前（1.785%, 1.785%）和术后（0.019%, 0.039%）抗凝组均无显著性差异（*P*=0.525, *P*=0.300）；引流管时间大于7 d和乳糜胸发生率在术前（5.357%, 1.785%）和术后（3.162%, 0.079%）抗凝组均无统计学差异（*P*=0.519, *P*=0.555）；皮下气肿和肺部感染发生率在术前（1.785%, 14.285%）和术后（1.581%, 6.324%）抗凝组均无显著性差异（*P*=0.989, *P*=0.085），见表 2。

**2:** 两组肺癌患者术后并发症分析 Postoperative complications of two groups of lung cancer patients

Characteristics	POST group (*n*=506)	PRE group (*n*=56)	*P*
Pulmonary embolism	1 (0.019%)	1 (1.785%)	0.525
Postoperation bleeding	2 (0.039%)	1 (1.785%)	0.300
Chylothorax	4 (0.079%)	1 (1.785%)	0.555
Chest tube retained > 7 d	16 (3.162%)	3 (5.357%)	0.519
Pulmonary infection	32 (6.324%)	8 (14.285%)	0.085
Subcutaneous emphysema	8 (1.581%)	1 (1.785%)	0.989

## 3 讨论

VTE包括深静脉血栓（deep venous thrombus, DVT）和肺动脉栓塞（pulmonary embolism, PE）两种同一疾病在不同部位、不同阶段的表现。在美国每年新发肺栓塞90万例，死亡29.6万人，是继感冒之后，引起丢失工作日丢失第2多的疾病，给社会造成的巨大的经济负担^[22-24]^。预防血栓栓塞和肺动脉栓塞是降低围手术期并发症和患者死亡的有效手段，也有助于术后的加速康复。

本研究主要目的是探索胸腔镜肺癌患者围手术期预防性抗凝的安全性及有效性，尽管国内外大量指南均推荐肿瘤外科手术患者从术前2 h-12 h开始预防性抗凝治疗，但这些证据的来源主要基于腹部及妇科肿瘤外科手术患者，目前胸外科尤其是肺癌手术预防性抗凝的证据很少，美国胸科医师学会（American College of Chest Physicians, ACCP）非骨科手术预防性抗凝指南在胸外科部分推荐意见中指出，由于胸外科研究数据较少，推荐意见是基于其它外科研究结果的推断得出。然而肺癌手术需要处理肺血管，手术难度大，风险高，术中渗血影响视野，容易损伤血管，造成严重后果，因此不能简单将其它肿瘤外科的经验用于肺癌手术。中国人与西方人的凝血功能存在较大差异，中国人的血小板量、凝血时间均显著低于高加索人。使用西方人群研究得出的预防性抗凝的方案不能直接用于中国人，然而目前尚无国人肺癌围手术期预防性抗凝安全性与有效性的数据。本研究56例肺癌患者中，1例患者术后当天出现胸腔内活动性出血，3 h引流量800 mL，紧急剖胸探查未发现明确出血不问，手术切口下方血凝块形成，考虑切口创面渗血，电凝止血后关胸，术后停用药物抗凝，患者正常康复出院。该患者术前2 h使用普通肝素（unfractionated heparin, UFH）预防性抗凝，发生出血时为术后8 h，术后抗凝药物尚未开始使用，且已超过UFH的半衰期1 h-6 h，因此术后切口渗血再次开胸手术是否与抗凝有关尚不明确。本组病例中，患者平均术中出血量，手术时间，术后引流管留置时间和术后住院时间与国内外文献报道的数据相比并无明显增高，可以推断，术前预防性抗凝可能是安全的。而术后抗凝的506例肺癌患者中，也有1例患者术后第二天出现胸腔引流量增多，呈血性，停用抗凝药后，出血停止且引流量减少，顺利拔管。但因抗凝药导致的术后出血发生率低，不应成为预防性用药的禁忌症。研究提示，术前用药患者术后72 h引流量显著高于术后用药患者，可能与凝血功能受到影响有关，但术后胸腔总引流量并无显著增加，提示围手术期预防性应用抗凝药物是安全性的。

本研究存在若干的局限性，首先是本研究为回顾性研究，肺栓塞及术后出血发生率较低，术前和术后用药组均仅出现1例，无法准确地反映围手术期药物预防性抗凝后肺栓塞的发生率及术后大出血的发生率。其次全组患者未术前常规行四肢深静脉血栓筛查，无法准确了解患者的基线资料，术后仅对有症状，怀疑存在VTE的患者行深静脉彩超及肺血管增强CT检查，可能遗漏部分无症状的VTE患者。第三，本研究的术中出血量、手术时间、术后住院时间等数据仅同国内外数据进行对比，存在较大差异，可比性不强，应谨慎得出结论。

本研究对肺癌患者围手术期预防性抗凝的初步探索，肺癌患者围手术期预防性抗凝是安全的，尚无确切证据证实围手术期预防性抗凝能够减少术后VTE的发生。需要通过大样本的前瞻性研究来证实中国人群肺癌术前预防性抗凝的安全性及有效性。
